# Increased Levels of CD107a and Intracellular Cytokines in IL-2 Stimulated PBMCs from Endometriosis Patients

**DOI:** 10.1155/2021/5760959

**Published:** 2021-09-27

**Authors:** R. Muharam, Ririn Rahmala Febri, Kevin Ardito Prabowo, Arleni Bustami, Indra G. Mansur

**Affiliations:** ^1^Division of Reproductive Endocrinology and Infertility, Department of Obstetrics and Gynecology, Faculty of Medicine, Universitas Indonesia, Jakarta 10430, Indonesia; ^2^Human Reproduction, Infertility, and Family Planning Research Center, Indonesian Medical Education and Research Institute (IMERI), Faculty of Medicine, Universitas Indonesia, Jakarta, 10430, Indonesia; ^3^Yasmin IVF Clinic, Dr. Cipto Mangunkusumo General Hospital, Jakarta 10430, Indonesia; ^4^Integrated Laboratory of Medical Faculty, Universitas Indonesia, Jakarta 10430, Indonesia; ^5^Master Program of Biomedical Sciences, Faculty of Medicine, Universitas Indonesia, Jakarta 10430, Indonesia

## Abstract

It has been postulated that the immune system is impaired in individuals with endometriosis, with attention directed to natural killer (NK) cells. Specifically, it has been hypothesized that altered numbers of peripheral NK cells in blood are associated with the presence of endometriotic lesions. This study aimed to evaluate the level of the peripheral NK cell surface marker CD107a in endometriosis in the presence of IL-2 stimulation. Peripheral blood mononuclear cells (PBMCs) were obtained from 7 women with endometriosis and 7 women without endometriosis. The PBMCs were divided into two groups and either treated with recombinant IL-2 or left untreated. The cytotoxic activity of the PBMCs toward target cells (K562) was evaluated. Then, both groups were cocultured for 4 days. The expressions of CD107a, TNF-*α*, and IFN-*γ* were determined using flow cytometry analysis. There was no difference in the expression of CD107a prior to IL-2 stimulation in PBMCs from women with endometriosis compared to those from women without endometriosis. However, we observed upregulation of the expression of the surface marker CD107a after treatment in the endometriosis group. In addition, there was a significant difference in CD107a expression in the endometriosis group before versus after stimulation with IL-2 (*p* < 0.01). We also found no difference in the production of TNF-*α* and IFN-*γ* before versus after treatment with IL-2 in either groups. The levels of CD107a were significantly enhanced in peripheral blood taken from women with endometriosis after treatment with IL-2.

## 1. Introduction

Endometriosis is one of the most common benign gynecological disorders and is histologically characterized by the presence of endometrial tissue outside the uterus [1]. Endometriosis causes dysmenorrhea, dyspareunia, pelvic pain, and infertility in women of reproductive age [2]. Despite its high prevalence and incapacitating symptoms, the exact pathogenic mechanisms of endometriosis are not completely understood. Immunological dysfunction in the peritoneal environment is believed to be a crucial factor in the development of endometriosis, as the displaced endometrial cells are able to avoid immune recognition [3]. Several studies have shown that inflammation contributes to the pathophysiology of the disease, mainly by altering the functions of immune cells and increasing the levels of proinflammatory cytokines in the peritoneal cavity, endometrium, and blood [4].

Natural killer (NK) cells are immune system components that play important roles in immunity against viruses and in tumor immune surveillance [5, 6]. These cells mainly circulate in the blood, where they account for ∼5–15% of circulating lymphocytes [7]. NK cells are granular lymphocytes that produce an abundance of inflammatory cytokines and release cytotoxic granular components to induce a killing mechanism against infected cells. NK cells participate in the pathogenesis of endometriosis by either allowing or inhibiting the survival, implantation, and proliferation of endometrial cells [3]. These mechanisms seem to be altered in endometriosis, indicating a decline in the cytotoxic function of NK cells that enable endometrial cells to attach to ectopic sites [7].

The cytoplasm of NK cells contains high concentrations of preformed cytotoxic granules that are uniquely designed to induce the death or target cells upon their release, eventually leading to the induction of apoptosis [8]. These granules are also known as secretory lysosomes and contain granzymes, perforin, and the lysosome-associated membrane proteins-1 (LAMP) 1 (CD107a), LAMP2/CD107b, and LAMP3/CD63 [9, 10]. In NK cells and cytotoxic T cells, CD107a is the most abundant protein in cytotoxic granules. Its cell surface expression has been described as a marker for cytotoxic T-cell degranulation and has been shown to be strongly upregulated following stimulation along with loss of perforin [11]. As degranulation occurs, secretory lysosomes are released, and CD107a is transported to the surfaces of NK cells, rendering them accessible for antibody binding and thus making it possible to identify NK cells that have been activated for deregulation. NK cells also secrete major inflammatory cytokines, such as tumor necrosis factor-*α* (TNF-*α*) and interferon-*γ* (IFN-*γ*) [12]. In many cases, the potent release of TNF-*α* and IFN-*γ* is sufficient for the effector function of NK cells in the absence of the perforin-dependent killing of other cells.

Altered numbers and decreased activity of cytotoxic NK cells have been found in the peripheral blood and peritoneal fluid of women with endometriosis, indicating that NK cells are functionally defective; however, the mechanism of this suppression remains unclear [7]. In addition, the levels of the cell surface cytotoxicity marker CD107a are also significantly reduced in this disease, which may contribute to the immune escape of menstrual endometrial fragments refluxed into the peritoneal cavity [13]. The levels of numerous cytokines, including interleukin-1*β* (IL-1*β*), IL-6, IL-8, TNF-*α*, and transforming growth factor-*β* (TGF-*β*), are higher in the peritoneal fluid of women with endometriosis than those in that of healthy control [14].

An enhanced understanding of the immune mechanisms occurring at sites of endometriotic lesion development should therefore provide invaluable insights into the disease pathogenesis [3]. CD107a assays are promising new tools for the characterization and possibly the immunotherapeutic use of endometriosis-specific NK cells [15]. Measurement of the activation of NK cells by detection of surface CD107a and detection of cytokine production is needed to enhance knowledge of the immune mechanisms involved in the development of endometriosis.

## 2. Materials and Methods

### 2.1. Subjects

Nine women (mean age: 35.11 ± 1.87 years) diagnosed with endometriosis at Dr. Cipto Mangunkusumo General Hospital were enrolled in this study from February to December 2017. Seven healthy women (mean age: 24.00 ± 0.53 years) were recruited as controls. None of the subjects had received any hormonal treatment for at least 3 months before the study. Informed consent was obtained from each subject, and the study was approved by the Ethics Committee of the Faculty of Medicine, University of Indonesia (46/UN2.F1/ETIK/2017).

### 2.2. Peripheral Blood Mononuclear Cell (PBMC) Isolation

Nine millilitre of blood was collected from the healthy volunteers and endometriosis women and transferred into tubes containing anticoagulant (heparin). PBMCs were prepared from fresh peripheral venous blood by Ficoll-Hypaque density gradient centrifugation (Sigma-Aldrich, St. Louis, MO). Each blood sample was diluted 1 : 1 (v/v) with RPMI 1640 medium and 9 ml of Ficoll-Hypaque. Then, the mixture was centrifuged in a centrifuge with a swing-out rotor at 400 ×g and 20°C for 30 min. PBMCs were collected from the interphase, washed in phosphate buffered saline (PBS), counted, and assessed for viability with trypan blue dye (0.2% (v/v) in PBS). Then, isolated PBMCs were aliquoted in cryovials and stored at −80°C until use or culture.

### 2.3. Cell Culture

PBMCs were cultured in complete medium (RPMI 1640 supplemented with 10% fetal bovine serum (FBS), 100 U/ml penicillin, 100 mg/ml streptomycin, and 1% amphotericin B; all from Sigma-Aldrich, St. Louis, MO) with a total volume of 5 ml in a humid environment at 37^o^C under 5% CO_2_. The cells were divided into 2 wells with 10^5^ PBMCs in each well. The first well was stimulated with 100 ng/ml interleukin-2 (IL-2) (Sigma-Aldrich) and incubated for 3 days (72 hours). The other was cultured without any stimulation. The cell line (K562) was added at day 3 and incubated for 24 hours. After cell culture, 1 *μ*l of GolgiStop (BD Biosciences, San Jose, US) containing monensin was added to every 6 ml of cell culture and was mixed thoroughly. Then, the mixture was incubated for another 4 hours before termination of the culture.

### 2.4. Cell Staining

Cells were stained for NK cell surface markers with CD56-fluorescein isothiocyanate (FITC) and CD107a-phycoerythrin (PE) antibodies (BD Biosciences) for 30 min at 4°C in 50 *μ*l of staining buffer (PBS + FBS 0.25%). The cells were washed twice with staining buffer. Following surface staining, the cells were resuspended thoroughly; then, 250 *μ*l of fixation/permeabilization solution was added, and the mixture was incubated for 20 min at 4°C. The cells were washed again two times in 1 ml of BD wash buffer. Next, allophycocyanin- (APC-) conjugated anti-TNF-*α* and anti-IFN-*γ* antibodies were used to stain the intracellular cytokines of PBMCs for 30 min at 4°C. Corresponding isotype controls were utilized as negative controls. After intracellular staining, the cells were washed twice with 1 ml of BD Perm/Wash buffer and resuspended in staining buffer prior to flow cytometric analysis.

### 2.5. Flow Cytometric Analysis

Three-color flow cytometry analysis was performed using a FACScan flow cytometer (Beckmann Coulter FC50), and the data were analyzed with CellQuest software (BD Biosciences). The fluorescence in channels FL1 (FITC), FL2 (PE), and FL3 (APC) was utilized to measure cell surface antigens and intracellular cytokines. Anti-CD56 (FITC) and anti-CD107a (PE) antibodies were used to identify the NK cell population [16]. All data are expressed as percentages.

### 2.6. Statistical Analysis

Statistical analysis and Pearson's correlation analysis were performed using Prism 5 software (GraphPad Software). The differences in the variables between women with endometriosis and women without endometriosis were assessed using independent-sample *t*-tests. Paired *t*-tests were used to compare the value of each variable before and after treatment. Pearson's correlation coefficient was used to determine the correlations between variables in both groups. A value of *p* < 0.05 was considered to indicate significance. All data are presented as mean ± SD unless otherwise indicated.

## 3. Results

Cell viability is defined as cells that reflect the number of cells that are negative for staining with trypan blue compared to the positive one. The total number of cells and the viability of the cells are shown in Table 1. The number of cells significantly decreased in the control group after cryopreservation; however, no significant difference was seen in the endometriosis group before and after cryopreservation (Table 1). We also observed a significant decrease in the viability of cells before and after cryopreservation (Table 1).

PBMCs were evaluated in the endometriosis group and the control group prior to IL-2 treatment. There was no difference in the expression of CD107a, TNF-*α*, or IFN-*γ* between the endometriosis group and the control group (Table 2).

**Figure 1:**
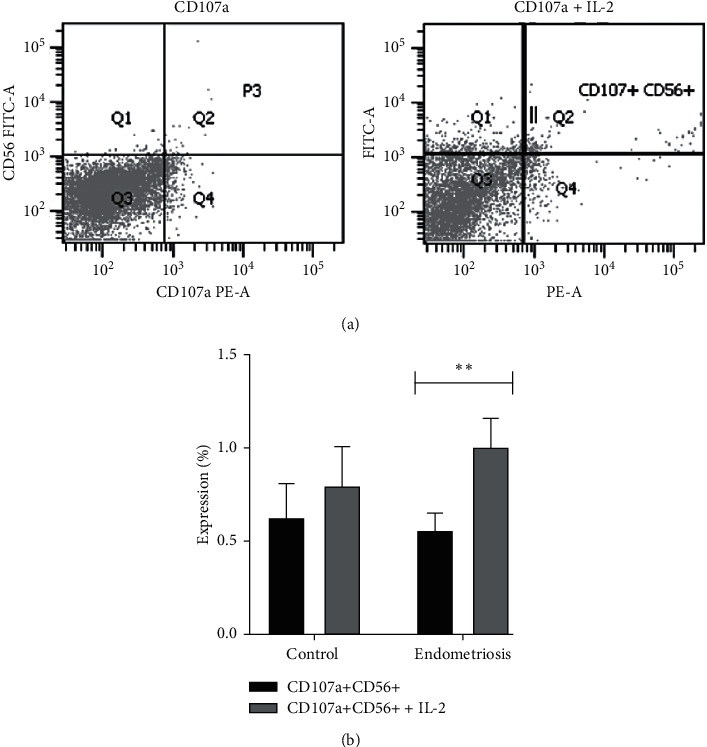
Upregulation of CD107a expression by IL-2 stimulation. (a) Flow cytometry analysis of the CD107a population without and with IL-2. (b) Comparison of CD107a expression before and after IL-2 stimulation in the control group and the endometriosis group. The differences before and after IL-2 stimulation were analyzed by paired *t*-test. ^*∗*^^*∗*^*p* < 0.01 was considered to indicate a significant difference.

To assess the effects of IL-2 on NK cell surface markers and intracellular cytokine levels, PBMCs were then cultured in the presence of IL-2. We observed upregulation of the expression of the surface marker CD107a after treatment (Figure 1(a)). In addition, there was a significant difference in CD107a expression in the endometriosis group before versus after stimulation with IL-2 (Figure 1(b)).

**Figure 2:**
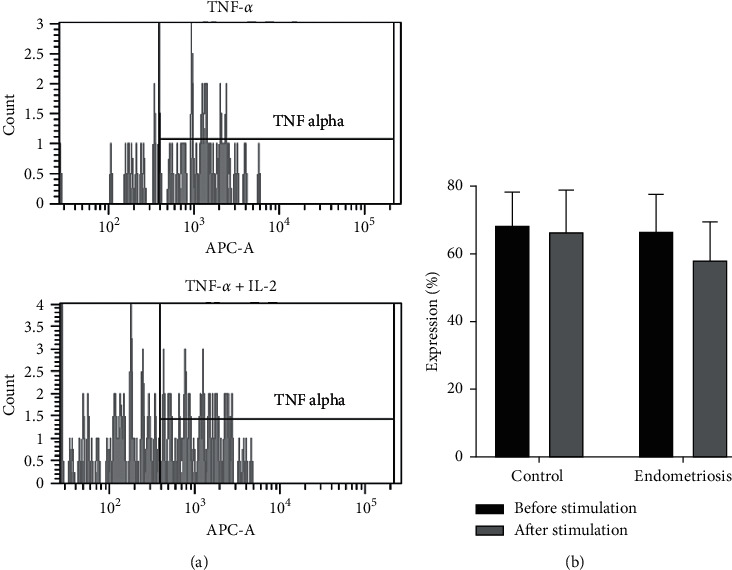
Expression of TNF-*α* in the presence and absence of IL-2. (a) Population of TNF-*α*+ cells as determined by flow cytometry analysis. (b) Expression of TNF-*α* before and after IL-2 stimulation. The differences before and after IL-2 stimulation were analyzed by paired *t*-test.

**Figure 3:**
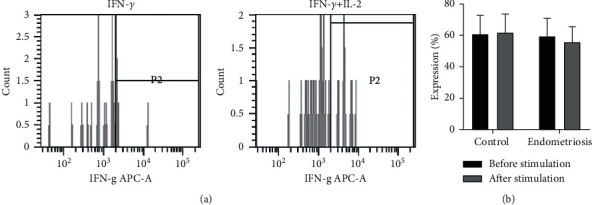
IFN-*γ* production in CD107a + CD56+ cells stimulated with IL-2. (a) Population of IFN-*γ*+ cells as determined by flow cytometry analysis. (b) Percentage of IFN-*γ* production in the endometriosis group and control group before and after IL-2 stimulation. The differences before and after IL-2 stimulation were analyzed by paired *t*-test.

Intracellular cytokines such as TNF-*α* and IFN-*γ* produced by peripheral NK cells were also assessed in this study. Upon stimulation, there were no significant differences in the expression of TNF-*α* and IFN-*γ* between women with endometriosis and women without endometriosis before and after treatment with IL-2 (Figures 2 and 3, respectively).

## 4. Discussion

It is well known that IL-2 induces the development and proliferation of NK cells [17]. In addition, IL-2 stimulation leads to activation and expansion of a CD56^bright^ population with high cytolytic activity against NK cell targets [18]. In this study, we found that the expression of NK cell cytotoxicity was not significantly different in the peripheral blood between the endometriosis and control groups. Interestingly, the levels of NK cell cytotoxic granules such as granzyme B, perforin, and CD107a were decreased in the peritoneal fluid of women with endometriosis [19]. We observed that, after treatment with IL-2, the expression of CD107a was significantly increased in the endometriosis group compared to the control group. A study by Martinovic et al. in metastatic melanoma patients showed that the expression of CD107a also increased after IL-2 treatment [20]. Furthermore, the combination of IL-2 and IL-12 exerts a more potent effect on increasing CD107a expression in NK cells [20]. In addition, Silva et al. reported that the expression of CD107a was significantly higher after IL-2 stimulation compared to that without stimulation in the epithelial ovarian cancer cell-free ascites [21]. These results indicate that NK cell cytotoxicity can be recovered via immunomodulatory treatment.

In addition, we also evaluated the production of TNF-*α* in C107a + CD56+ cells in the PBMC from women with endometriosis and women without endometriosis. We found that there was no difference in TNF-*α* production between the groups. It is known that TNF-*α* is a major proinflammatory cytokine that has been implicated in endometriosis pathogenesis [22]. TNF-*α* also plays a role in endometriotic tissue-induced stimulation of the expression of matrix metalloproteinases, which actively participate in the invasion and matrix remodeling of endometriotic lesions. A study by Wang et al. reported that peritoneal fluid of endometriosis patients contains an increased number of activated macrophages that secrete local products with important angiogenic properties, including TNF-*α* [23]. This result is in agreement with recent studies showing that the concentrations of TNF-*α* are significantly higher in the peritoneal fluid of women with endometriosis than those in that of women without endometriosis [24]. TNF-*α* secreted by activated macrophages has potent inflammatory, cytotoxic, and angiogenic properties and is involved in the development of endometriosis [25]. In this study, we also reported that TNF-*α* expression showed no difference in the endometriosis and control groups after IL-2 stimulation.

Furthermore, this study demonstrated that there was no difference in the expression of IFN-*γ* in CD107a + CD56+ cells between women with endometriosis and those without endometriosis. Although an increase in the CD107a + CD56+ cell percentage after treatment with IL-2 was observed in the endometriosis group, the amount of IFN-*γ* produced by CD107a + CD56+ cells was slightly decreased in this group. On the other hand, a study by Tarokh et al. demonstrated a decreased level of IFN-*γ* in the endometriosis group compared to the control group [26]. Likewise, Gmyrek et al. showed that the production of IFN-*γ* was reduced in peripheral lymphocytes of endometriosis [27]. IFN-*γ*-producing NK cells have been found to have low cytotoxicity, whereas cytotoxic NK cells express low levels of IFN-*γ* [28]. In addition, IFN-*γ* is recognized to induce resistance of target cells to NK cytolysis. The cytolytic function of NK cells is controlled by a number of activating and inhibitory receptors, and target cell recognition by activating receptors such as NKG2D leads to the production of IFN-*γ* [29].

One of the limitations of this study is the small number of respondents. The number of respondents was too small to generalize the cytotoxic activity of NK cells in endometriosis. However, further study in larger sample size could have generated more accurate results.

## 5. Conclusions

In conclusion, stimulation with IL-2 increased the percentages of CD107a + CD56+ cells among PMBCs from women with endometriosis but did not increase the levels of the cytokines produced by these cells, TNF-*α* and IFN-*γ*. TNF-*α* and IFN-*γ* secretions and cytotoxicity are regarded as two distinct functions of NK cells with little synergy as a result of the early association of the two functions with distinct subsets of NK populations.

## Figures and Tables

**Table 1:** The number and viability of cells before and after cryopreservation in endometriosis and control groups.

Variables	Before cryopreservation	After cryopreservation	*p* value
The number of cells (n)			
Control group	6.41 × 10^6^ ± 5.22 × 10^5^	4.81 × 10^6^ ± 4.23 × 10^5^	0.003^*∗*^
Endometriosis group	5.41 × 10^6^ ± 6.17 × 10^5^	3.54 × 10^6^ ± 6.62 × 10^5^	NS
The viability of cells (%)			
Control group	99.16 ± 0.23	90.62 ± 2.52	0.017^*∗*^
Endometriosis group	97.56 ± 1.05	90.90 ± 1.65	0.011^*∗*^

The values are expressed as mean ± SE. ^∗^*p* < 0.05 was considered to indicate statistical significance. NS, not significant.

**Table 2:** Comparison of surface markers and intracellular cytokines between the endometriosis and control groups.

Variables	Control group (*n* = 7)	Endometriosis group (*n* = 9)	*p* value
CD107a + CD56+	0.63 ± 0.18	0.56 ± 0.09	NS
TNF-*α*	68.63 ± 9.73	66.92 ± 10.79	NS
IFN-*γ*	60.91 ± 11.80	59.64 ± 11.41	NS

The values are expressed as mean ± SE. *p* < 0.05 was considered to indicate statistical significance. NS, not significant.

## Data Availability

Data are not freely available for third-party use as this is not covered by the ethics approval for this project.
